# A comparative study of EEG functional and effective connectivity patterns in children with learning difficulties during reading and math tasks

**DOI:** 10.3389/fnins.2025.1612884

**Published:** 2025-08-18

**Authors:** Haiyan Liu, Huimin Liu

**Affiliations:** ^1^School of Psychology, Hainan Normal University, Haikou, China; ^2^Adolescent Psychological Development and Education Center of Hainan, Haikou, China

**Keywords:** learning difficulties, EEG, brain network connectivity, wPLI, DTF

## Abstract

**Introduction:**

This study utilized electroencephalography (EEG) to compare brain functional and effective connectivity patterns in children with reading difficulties (RD) and math difficulties (MD) during specific tasks. The aim was to identify neurophysiological distinctions between these two learning disorders, which often exhibit high comorbidity.

**Methods:**

Data from a publicly available dataset of 28 children (11 RD, 17 MD) aged 7–13 years were analyzed. Functional connectivity was quantified using the weighted Phase Lag Index (wPLI), and effective connectivity was assessed with the Directed Transfer Function (DTF).

**Results:**

Functional connectivity analysis revealed significant group differences. The RD group showed significantly higher beta band synchronization in the right temporal lobe compared to the MD group. Conversely, the MD group exhibited significantly greater connectivity in the frontal lobe's delta band and the parietal lobe's theta band. However, no statistically significant differences were observed between the groups regarding effective connectivity.

**Discussion:**

These findings highlight specific task-related brain functional connectivity differences between reading and math learning difficulties, suggesting potential compensatory mechanisms in RD and cognitive control challenges in MD. The lack of significant effective connectivity findings may be attributed to the small sample size, which is a key limitation of the study. This research emphasizes the need for larger samples, refined task designs, and multimodal neuroimaging in future studies.

## 1 Introduction

Learning Disorders (LDs) represent a significant challenge in education and critically impact social equity and population quality. Defined by one or more impairments or developmental delays in specific academic domains such as reading or mathematics (Prior, 2022), these disorders affect a substantial portion of the global child population. Epidemiological data indicate that ~7% of children worldwide are affected by Reading Difficulties (RD), while the prevalence of Math Difficulties (MD) ranges from 5% to 8% (Geary, 2004; Yang et al., 2022). This implies that one in 20 children faces significant challenges in reading or mathematical abilities. Beyond academic underperformance, these disorders are closely associated with mental health issues, including anxiety and low self-esteem (Georgiou and Parrila, 2023; Majid, 2024).

Notably, RD and MD exhibit a high comorbidity rate, averaging around 40%, with an average prevalence of 37% for one disorder when the other is present (Wilson et al., 2015). Due to variations in diagnostic criteria and task definitions across different regions, the overall comorbidity rate ranges from 11% to 70% (Moll et al., 2021). This consistently high comorbidity suggests that these two cognitive abilities may, at least partially, rely on shared neural networks (Plomin and Kovas, 2005). Neuroimaging studies further support this view, demonstrating that while RD and MD involve functionally specific brain regions, they also share overlapping areas (Ashkenazi et al., 2013). For instance, functional magnetic resonance imaging (fMRI) studies have revealed that the left hemisphere language network and bilateral parietal lobes (particularly the intraparietal sulcus, IPS) are activated during both reading and math tasks (Wang et al., 2020). The angular gyrus, acting as a hub for multimodal information integration, may coordinate semantic extraction and quantity-symbol mapping through distinct neural oscillation patterns (Dehaene et al., 2003). Furthermore, a longitudinal study revealed that improvements in reading ability significantly predict progress in mathematical performance among children with learning disabilities, further confirming the close behavioral association between RD and MD (Erbeli et al., 2021).

The rapid advancements in neuroscience have recently prompted researchers to delve deeper into the underlying brain mechanisms of these disorders, aiming to uncover their intrinsic patterns through the study of neural activity (Martínez-Briones et al., 2025). Among these, neural oscillations, as dynamic patterns of brain activity, offer a new perspective for understanding the neurobiological basis of reading and math disorders (Cao et al., 2022; Handayani et al., 2018). Neural oscillations are categorized into different frequency bands, with each band associated with specific cognitive functions and brain states (see Table 1).

**Table 1:** EEG frequency ranges (Hz): cognitive roles and links to learning.

**Frequency band (Hz)**	**Associated cognitive functions/states**	**Relevance to learning difficulties**
Delta (δ) 0.5–4	Deep sleep, relaxation, interference inhibition, internal focus, attention, functional cortical differentiation.	Excessive delta and theta waves in children with learning difficulties may indicate inefficient cognitive processing. Enhanced delta phase synchrony in math experts.
Theta (θ) 4–8	Attention, memory formation, emotional processing.	Theta band power is negatively correlated with intelligence in children. Plays a role in cognitive functions like working memory.
Alpha (α) 8–13	Awake relaxation, disengagement from external stimuli, inhibition of irrelevant visual input, semantic integration, attention, arousal.	Increased alpha band activity during reading tasks may inhibit irrelevant visual input. Children with learning difficulties may have deficits in sensory gating.
Beta (β) 13–30	Attention, motor control, cognitive processing, maintenance of symbolic representation in working memory, computational reasoning, executive control.	Beta band plays an important role in the frontoparietal network during math tasks. Children with Math Difficulties (MD) may have abnormal frontoparietal connectivity.

The delta band (0.5–4 Hz) is typically associated with deep sleep and relaxation. However, during cognitive tasks, increased delta band activity in frontal regions may be related to inhibiting interference and promoting internal focus, potentially by regulating networks that should be inactive during task execution (Harmony, 2013; Knyazev, 2012). In children with learning disabilities, excessive slow waves (such as delta and theta waves) may indicate inefficient cognitive processing (Fonseca et al., 2006; Martínez-Briones et al., 2025). The theta band (4–8 Hz) is closely linked to attention, memory formation, and emotional processing. Research has found that theta band power is negatively correlated with intelligence in children (Martínez-Briones et al., 2025). Theta band oscillations also play a crucial role in cognitive functions, including working memory (Tian et al., 2025). The alpha band (8–13 Hz) typically appears during awake relaxation, particularly prominent in parietal and occipital regions. Increased alpha band activity reflects healthy relaxed alertness and disengagement from external stimuli (Martínez-Briones et al., 2025; Sharma and Singh, 2015). In cognitive tasks, alpha band suppression (event-related desynchronization, ERD) is associated with increased attention levels and arousal (Martínez-Briones et al., 2025). During reading tasks, increased alpha band activity in posterior brain regions (e.g., temporal and occipital lobes) is considered a key mechanism for inhibiting irrelevant visual input and optimizing semantic integration (Liljefors et al., 2024; Viswanathan et al., 2023). The beta band (13–30 Hz) is associated with attention, motor control, and higher-level cognitive processing. In mathematical tasks, beta band oscillations play an important role in the frontoparietal network, helping maintain symbolic representation in working memory and supporting computational reasoning (Zioga et al., 2023). Low beta rhythms in the parietal cortex are considered a “buffer zone” for working memory, capable of receiving and integrating executive commands from the prefrontal cortex (Gelastopoulos et al., 2019). In children with developmental coordination disorder, abnormal beta oscillatory dynamics are associated with poor procedural learning (Lum et al., 2025).

Cognitive functions of the human brain are not merely products of localized brain activity; they fundamentally rely on the integration of information among distributed brain regions (Bastos and Schoffelen, 2016). Therefore, in-depth investigation of functional and effective connectivity is crucial for understanding the neural architecture of RD and MD. While previous studies have identified changes in local brain activity or overall spectral power in children with RD and MD (Ashkenazi et al., 2013; Moll et al., 2016), task-driven, frequency-specific comparative studies from a dynamic brain network perspective, particularly regarding synchrony and directional characteristics of connections, remain limited.

This study aims to systematically compare the frequency-specific features of brain functional and effective connectivity in children with RD during reading tasks vs. children with MD during math tasks, using EEG combined with wPLI and DTF. We hypothesize that, within their respective tasks, the brain network connectivity patterns of children with RD and MD, including synchrony and directed information flow, will exhibit distinguishable characteristics that reflect their specific cognitive challenges. Specifically, we expect that in reading tasks, children with RD may show abnormal functional connectivity in brain regions related to language processing, such as alpha or beta band activity in the right temporal lobe, which might reflect compensatory mechanisms. Conversely, in math tasks, children with MD may exhibit abnormal functional connectivity in frontoparietal networks associated with numerical processing and working memory, such as delta band activity in the frontal lobe or theta band activity in the parietal lobe. These oscillatory “directional codes” may reveal commonalities and distinctions between the two learning disorders, providing neurophysiological evidence for understanding their underlying mechanisms and establishing a neuromarker framework for early identification and intervention.

## 2 Materials and methods

### 2.1 Dataset

**Figure 1:**
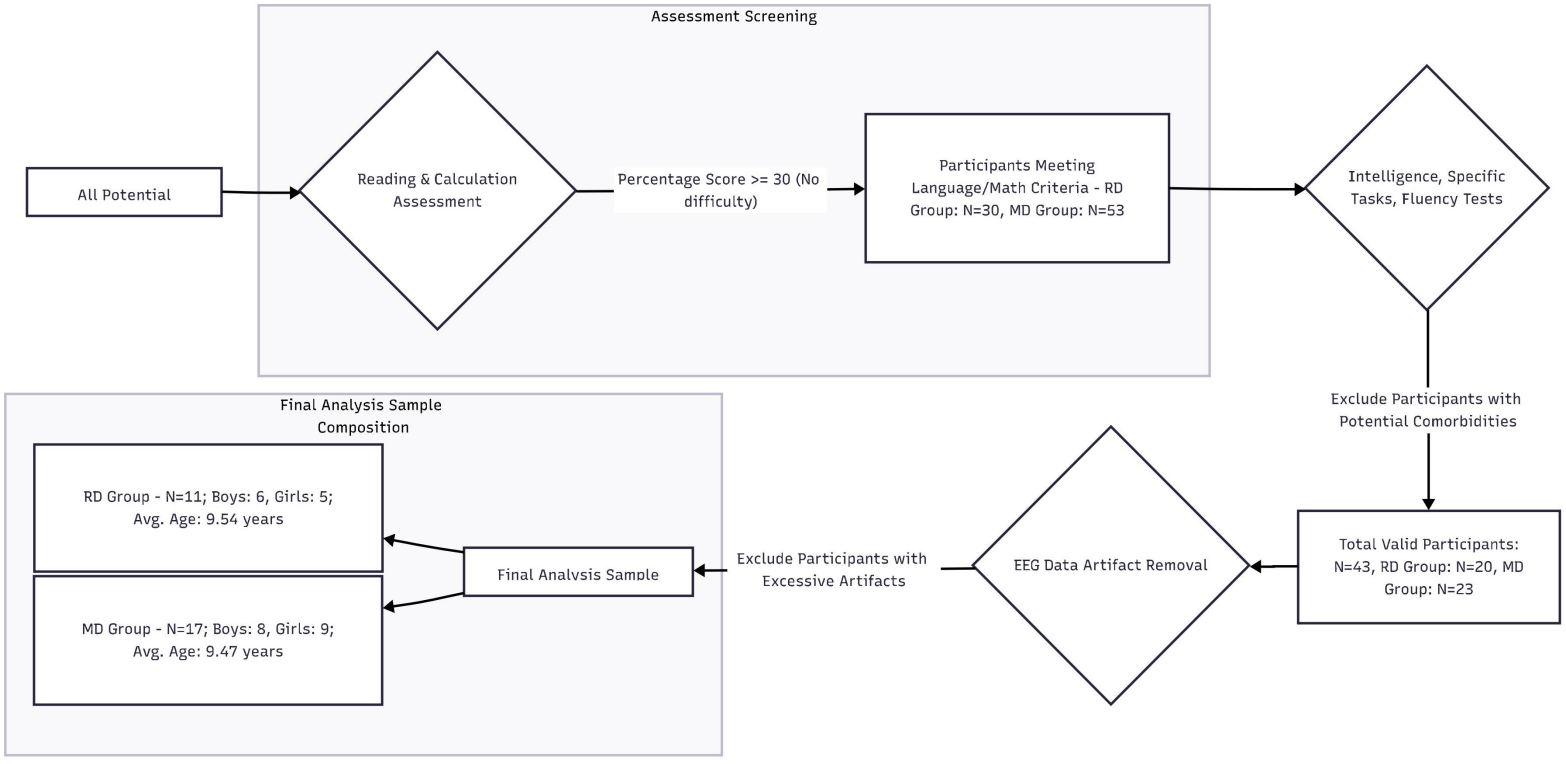
Subject screening process.

The dataset used in this study is publicly available through the Mendeley Data repository under the project titled “EEG data and psychometric results from children with learning difficulties” (Corona-Gonzalez et al., 2024). Initiated by the Tecnológico de Monterrey, the project collected psychometric and task-based electroencephalography (EEG) data from 104 children aged 7 to 13 who demonstrated low academic performance, with the aim of assessing their reading and mathematical abilities. Initially, the project evaluated a range of cognitive and behavioral metrics including reading fluency, spelling, mathematical computation, attention, and intelligence quotient (IQ), in order to determine each child's primary learning difficulty. Based on these assessments, the participants were classified into two groups: children with Reading Difficulties (RD, *n* = 54) and children with Math Difficulties (MD, *n* = 50). EEG data from both groups were collected using a 32-channel system while participants engaged in reading or math-related tasks. For the present study, a subset of data from 28 children with learning difficulties was selected for further analysis (RD group: *n* = 11; MD group: *n* = 17). The participant selection process is illustrated in Figure 1.

### 2.2 Data pre-processing

Offline EEG data analysis was conducted using MATLAB R2023b and EEGLAB 2025.0.0. EEG signals were downsampled to 200 Hz and pre-processed using a bandpass filter (1–45 Hz). The continuous EEG was segmented into 2-s epochs. Independent Component Analysis (ICA) was applied to remove artifacts related to eye movements and muscle activity, and segments exceeding ±200 μV were excluded as abnormal. Data were then re-referenced to the average of all electrodes, and defective channels were interpolated using spherical spline interpolation. The pre-processed data were subsequently used for both functional and effective connectivity analyses.

### 2.3 Functional connectivity

In EEG analysis, functional connectivity refers to the statistical dependencies between spatially distinct brain regions over time (Bastos and Schoffelen, 2016). Unlike effective connectivity, it does not imply causality, but rather identifies “which areas are synchronously or correlatively active.”

This study employed the Weighted Phase Lag Index (wPLI) to quantify the degree of phase synchronization between EEG time series from different brain regions (Vinck et al., 2011). The standard Phase Lag Index (PLI), introduced by Stam et al. (2007), measures asymmetry in phase difference distributions, ignoring zero-phase lag connections to minimize volume conduction artifacts. wPLI extends this by assigning greater weight to phase differences with higher magnitude, enhancing sensitivity to genuine phase synchronization. Compared to other measures such as coherence or PLI, wPLI is more robust to noise and better at reducing spurious connectivity caused by volume conduction (Hardmeier et al., 2014; Stam et al., 2007). The calculation involves the following steps:

For two signals *x* and *y*, their instantaneous phases ϕ_*x*_(*t*) and ϕ_*y*_(*t*) are calculated via the Hilbert transform:


(1) $$\begin{array}{l} \phi_{x}\left(t\right)=\arg\left(x\left(t\right)+j\cdot H\left\{x\left(t\right)\right\}\right) \end{array}$$

(2) $$\begin{array}{l} \phi_{y}\left(t\right)=\arg\left(y\left(t\right)+j\cdot H\left\{y\left(t\right)\right\}\right) \end{array}$$

Calculate the phase difference:


(3) $$\begin{array}{l} \phi \left(t\right)=\phi_{y\left(t\right)}-\phi_{x\left(t\right)} \end{array}$$

Extract the imaginary part of the phase difference:


(4) $$\begin{array}{l} \text{Im}\left(e^{i\varphi \left(t\right)}\right)=\sin\left(\phi \left(t\right)\right) \end{array}$$

The wPLI is computed as:


(5) $$\begin{array}{c} \text{wPLI}=\frac{\left|\sum_{\text{t=1}}^{\text{T}}\text{Im}\left({\text{S}}_{\text{xy}}\left(\text{t}\right)\right)\right|}{\sum_{\text{t=1}}^{\text{T}}\left|\text{Im}\left({\text{S}}_{\text{xy}}\left(\text{t}\right)\right)\right|} \end{array}\;$$

Where t denotes the total number of time points. The wPLI ranges from 0 to 1, where values near 0 indicate no consistent phase lead or lag, and values approaching 1 indicate high phase consistency across trials.

### 2.4 Effective connectivity

In contrast to functional connectivity, effective connectivity seeks to uncover causal interactions or the directional flow of information between brain regions (Cao et al., 2022). It addresses the question of “who influences whom.”

This study utilized the Directed Transfer Function (DTF) to examine directional neural interactions. Introduced by Kaminski and Blinowska (2017), DTF is grounded in the Granger Causality Analysis framework and estimates directional influence by fitting a Multivariate Autoregressive (MVAR) model to multichannel EEG time series (Astolfi et al., 2006; Kaminski and Blinowska, 2017). The procedure involves:

Fitting an MVAR model:


(6) $$\begin{array}{l} x_{i}\left(n\right)=-\overset{M}{\underset{j=1}{\sum }}\overset{p}{\underset{k=1}{\sum }}a_{ij}\left(k\right)x_{j}\left(n-k\right)+u_{i}\left(n\right) \end{array}$$

where *x*_*i*_(*n*) is the EEG signal from channel *i* at time *n*, *M* is the number of channels, *p* is the model order, *a*_*ij*_(*k*) are model coefficients, and *u*_*i*_(*n*) is Gaussian white noise.

Computing the transfer function in the frequency domain:


(7) $$\begin{array}{l} \text{H}{\left(f\right)}_{ij}=-\frac{1}{1+\overset{p}{\underset{k=1}{\sum }}a_{ii}\left(k\right)e^{-i2\pi fk\Delta t}}\overset{p}{\underset{m=1}{\sum }}a_{ij}\left(m\right)e^{-i2\pi fm\Delta t} \end{array}$$

where Δ*t* is the sampling interval.

Computing DTF from channel *j* to *i* at frequency *f*:


(8) $$\begin{array}{l} {\text{DTF}}_{ij}\left(f\right)=\frac{\left|H{\left(f\right)}_{ij}\right|}{\sqrt{\overset{M}{\underset{k=1}{\sum }}|H{\left(f\right)}_{ik}{|}^{2}}} \end{array}$$

This normalization ensures that the squared DTFs from all input channels to a given output channel sum to 1 at each frequency. DTF thus provides a frequency-specific estimate of how one EEG channel influences another, supporting studies of task-related information flow and functional brain network construction (Ding et al., 2000; Kaminski and Blinowska, 1991).

To preserve directionality, which can be obscured by averaging across regions, we calculated DTF only within individual hemispheres. EEG signals from all channels in the left and right frontal [LF [FP1, F3, F7], RF [FP2, F4, F8]], temporal [LT [T7, TP9], RT [T8, TP10]], parietal [LP [CP1, CP5, P3, P7], RP [CP2, CP6, P4, P8]], and occipital [LO [O1], RO [O2]] regions were averaged separately. Given that the MVAR model assumes signal stationarity, we treated the EEG data as quasi-stationary by segmenting it accordingly.

### 2.5 Statistical analysis

All statistical analyses were conducted using MATLAB (R2023b). Independent-samples *t*-tests were performed to examine group differences in wPLI and DTF values between the RD and MD groups. To control for potential confounding variables, we included IQ, attention, gender, and test scores as covariates in our statistical model.

## 3 Results

This study conducted a comparative analysis of EEG connectivity patterns in 28 children with learning disabilities during reading and mathematical tasks. Our primary objective was to identify potential differences in functional connectivity, quantified by the weighted Phase Lag Index (wPLI), and directed information flow, assessed via the Directed Transfer Function (DTF), between children with Reading Difficulties (RD) performing reading tasks and children with Math Difficulties (MD) engaged in mathematical tasks.

### 3.1 Functional connectivity analysis

The functional connectivity analysis using wPLI revealed three statistically significant differences (p < 0.05) between the RD and MD groups. Specifically, in the frontal lobe's delta band, the MD group demonstrated significantly higher wPLI values (M ± SD = 0.342 ± 0.042) compared to the RD group (M ± SD = 0.319 ± 0.036; *t* = −2.130, *p* = 0.038). Conversely, the RD group exhibited significantly greater wPLI values (M ± SD = 0.208 ± 0.117) than the MD group (M ± SD = 0.148 ± 0.056) in the right temporal lobe's beta band (*t* = 2.305, *p* = 0.025). Furthermore, the MD group displayed significantly higher wPLI values (M ± SD = 0.261 ± 0.024) in the parietal lobe's theta band compared to the RD group (M ± SD = 0.247 ± 0.021; *t* = −2.253, *p* = 0.029). The detailed *t*-test results for wPLI across various brain regions are presented in Table 2.

**Table 2:** wPLI *t*-test results for each brain region.

**Region**	**Band**	**M**±**SD**		***t*-value**	***p*-value**
		**RD**	**MD**		
Frontal	Delta	0.319 ± 0.036	0.342 ± 0.042	−2.130^*^	0.038
	Theta	0.254 ± 0.035	0.256 ± 0.035	−0.271	0.787
	Alpha	0.227 ± 0.029	0.220 ± 0.039	0.656	0.515
	Beta	0.142 ± 0.026	0.156 ± 0.036	−1.678	0.100
Left temporal	Delta	0.349 ± 0.111	0.313 ± 0.082	1.285	0.205
	Theta	0.253 ± 0.101	0.246 ± 0.070	0.284	0.777
	Alpha	0.230 ± 0.080	0.229 ± 0.065	0.044	0.965
	Beta	0.199 ± 0.102	0.161 ± 0.074	1.519	0.135
Right temporal	Delta	0.341 ± 0.092	0.345 ± 0.120	−0.146	0.884
	Theta	0.257 ± 0.079	0.238 ± 0.063	0.965	0.339
	Alpha	0.218 ± 0.070	0.185 ± 0.071	1.670	0.101
	Beta	0.208 ± 0.117	0.148 ± 0.056	2.305^*^	0.025
Parietal	Delta	0.341 ± 0.051	0.328 ± 0.048	0.961	0.341
	Theta	0.247 ± 0.021	0.261 ± 0.024	−2.253^*^	0.029
	Alpha	0.232 ± 0.024	0.240 ± 0.022	−1.194	0.238
	Beta	0.178 ± 0.065	0.157 ± 0.034	1.454	0.152
Occipital	Delta	0.346 ± 0.087	0.337 ± 0.131	0.268	0.790
	Theta	0.259 ± 0.092	0.267 ± 0.090	−0.292	0.772
	Alpha	0.236 ± 0.047	0.238 ± 0.074	−0.109	0.914
	Beta	0.186 ± 0.100	0.152 ± 0.067	1.430	0.159

^*^p < 0.05, ^**^p < 0.01, ^***^p < 0.001.

### 3.2 Effective connectivity analysis

**Figure 2:**
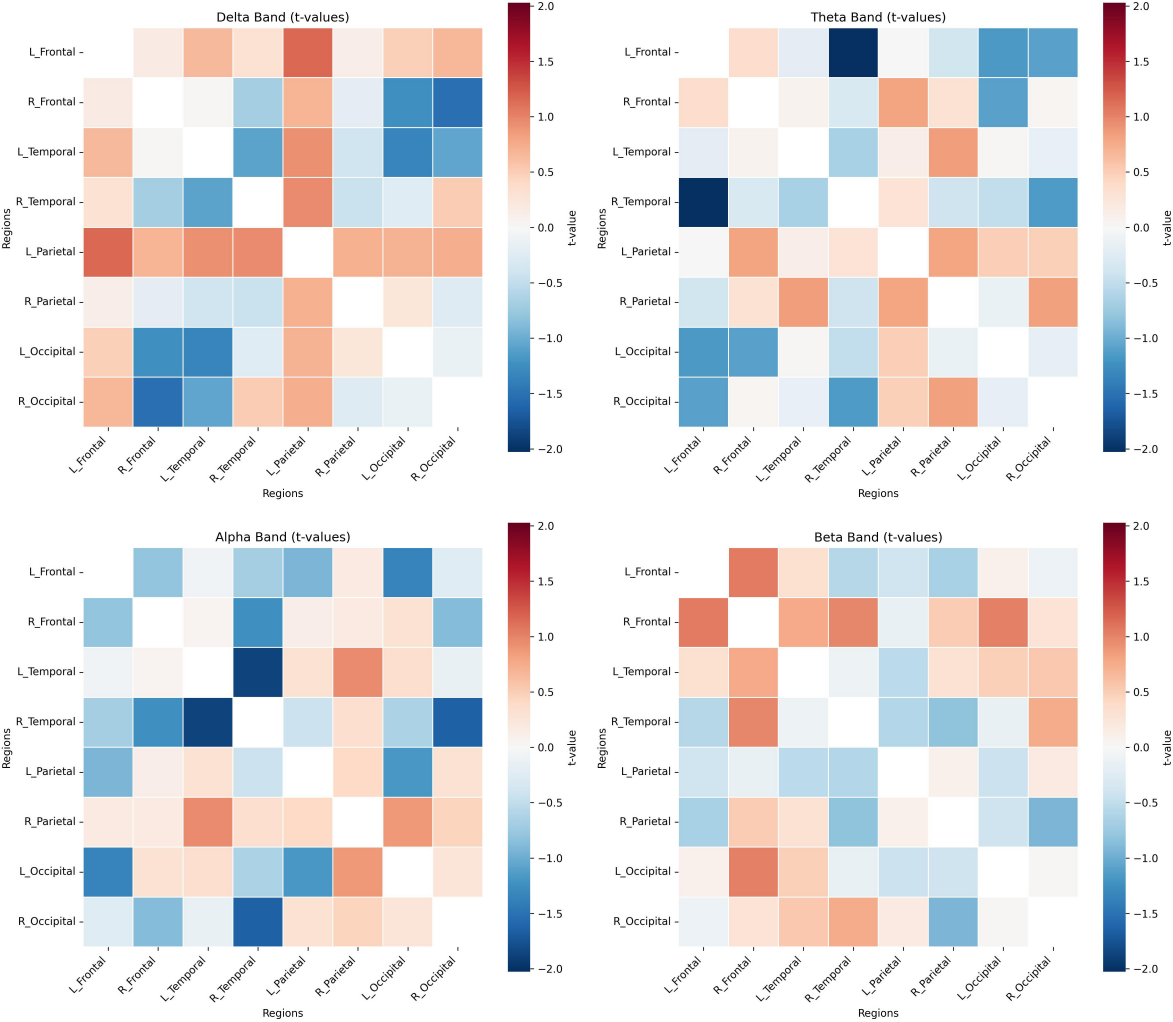
Statistical comparisons of interhemispheric effective connectivity between the RD and MD groups (color bars represent *t*-values).

In contrast to the functional connectivity findings, the analysis of directed information flow using DTF did not reveal any statistically significant differences between the RD and MD groups across any of the explored cross-regional connections or frequency bands (*p* > 0.05). All obtained *p*-values consistently exceeded 0.05, indicating that, within the current study design and given the sample size, no statistically significant distinctions in directed connectivity were observed between the groups. A heatmap illustrating the *t*-test results for each frequency band can be found in Figure 2.

## 4 Discussion

This study aimed to compare electroencephalography (EEG) functional and effective connectivity patterns in children with Reading Disability (RD) during reading tasks vs. children with Math Disability (MD) during mathematical tasks. Our findings revealed three significant group differences in functional connectivity but no statistically significant distinctions at the effective connectivity level. These results offer new perspectives on the neurophysiological underpinnings of both learning disorders and prompt deeper consideration of task-specific brain network dynamics.

### 4.1 Neurocognitive interpretation of functional connectivity differences

The significant differences observed in functional connectivity provide neurophysiological clues regarding the cognitive processing characteristics of RD and MD children in their respective tasks, aligning with the hypotheses presented in the introduction concerning abnormalities in specific brain regions and frequency bands.

The MD group exhibited significantly higher connectivity in the frontal lobe's delta band compared to the RD group. Delta band activity in cognitive tasks, particularly its increase in frontal regions, is generally considered to reflect the inhibition of interference, internal focus, and the regulation of inactive networks (Harmony, 2013; Knyazev, 2012). The higher frontal delta band connectivity in MD children during math tasks might indicate a greater need for cognitive control to suppress irrelevant information when solving mathematical problems, or it could reflect a specific mode of frontal lobe involvement in response to high cognitive load. This finding aligns with the commonly observed deficits in working memory and executive functions among MD children (Moll et al., 2016; Peng and Fuchs, 2016), suggesting that their frontal networks might be overactivated or inefficient in maintaining task focus or coping with computational challenges. For example, Poikonen et al. (2024) found that math experts exhibited enhanced frontoparietal delta phase synchrony when processing complex mathematical tasks (Poikonen et al., 2024). This contrasts with the trend of increased frontal delta band connectivity in MD children observed in our study, potentially implying a qualitative difference in the delta band synchrony patterns of MD children compared to typically developing children or experts; it may not be a sign of efficient processing, but rather an indication of increased cognitive effort.

The RD group showed significantly higher connectivity in the right temporal lobe's beta band compared to the MD group. The beta band is generally associated with attention, motor control, and higher-level cognitive processing, playing a particularly important role in maintaining symbolic representations in working memory and supporting computational reasoning (Gelastopoulos et al., 2019; Zioga et al., 2023). The right temporal lobe is often considered an auxiliary region to the left hemisphere language network in language processing, potentially involved in semantic integration or non-linguistic information processing (Fraga González et al., 2018; Shaywitz and Shaywitz, 2008). The enhanced right temporal beta band connectivity in RD children during reading tasks may reflect a compensatory strategy. When the left hemisphere's language processing networks (e.g., temporoparietal regions) are functionally impaired, the right hemisphere might be more activated to assist with reading tasks, such as processing visual-spatial information or non-lexical semantic cues (Fraga González et al., 2018; Yang et al., 2025). This right-hemisphere involvement could represent an adaptive reorganization of the brain to maintain task performance in the face of reading difficulties. This finding is partially consistent with Yang's (2025) research on functional connectivity patterns in children with reading disabilities, which also suggested that increased frontal-central connectivity might reflect compensatory mechanisms.

The MD group exhibited significantly higher connectivity in the parietal lobe's theta band compared to the RD group. The theta band is closely associated with attention, memory formation, and emotional processing, with its power negatively correlated with children's intelligence (Martínez-Briones et al., 2025; Tian et al., 2025). The parietal lobe plays a central role in numerical processing and spatial cognition, with the intraparietal sulcus (IPS) being a key region for quantity processing (Dehaene et al., 2003; Rotzer et al., 2009). The enhanced parietal theta band connectivity in MD children during math tasks may reflect an over-reliance on or inefficiency of the parietal regions during numerical processing. This could be related to the difficulties MD children face in numerical representation, arithmetic fact retrieval, and spatial working memory (Kucian and von Aster, 2015; Peng and Fuchs, 2016). Higher theta band activity might indicate that these children need to invest more cognitive resources to sustain attention and working memory during math tasks, or it could reflect functional impairments within their parietal networks when processing mathematical information. Martínez-Briones et al. (2025) also found that children with learning disabilities exhibit excessive delta and theta band power, which may be a sign of inefficient cognitive processing, consistent with the increased parietal theta band connectivity observed in MD children in our study.

### 4.2 Considerations for non-significant differences in effective connectivity

In contrast to the significant findings in functional connectivity, this study did not detect statistically significant differences in effective connectivity between the RD and MD groups. This outcome could be attributed to a combination of factors. The measurement of effective connectivity, particularly DTF based on MVAR models, is more sensitive to data quality, sample size, and the fulfillment of model assumptions (Mehdizadehfar et al., 2023; Seth et al., 2015). The relatively small sample size in this study (11 in the RD group, 17 in the MD group) might have led to insufficient statistical power, making it difficult to capture genuinely existing, yet potentially more subtle, differences in directed information flow. Mehdizadehfar et al. (2023) emphasized that connectivity estimates from small sample sizes may be less robust and exhibit greater variability, providing a methodological explanation for the non-significant results observed in this study. Furthermore, the RD group performed a reading task while the MD group performed a mathematical task. This difference in task type might have masked learning disability-specific effective connectivity patterns. Different tasks activate distinct cognitive processes and neural networks, complicating a direct comparison of effective connectivity between the two groups under different tasks. Even if potential differences in directed information flow exist, they might have been confounded by task effects. Therefore, the absence of significant effective connectivity differences does not necessarily mean that the information flow directions are identical in both disorders, but rather suggests the need for more refined task designs and larger sample sizes to uncover these potential differences.

### 4.3 Clinical implications and future directions

This study provides novel insights into the neural mechanisms of RD and MD children through EEG connectivity analysis. The observed functional connectivity differences in the frontal delta band, right temporal beta band, and parietal theta band may serve as potential neurobiological markers for distinguishing between these two learning disorders. These findings offer a theoretical basis for developing more targeted neuro-modulation intervention strategies. For instance, addressing the abnormal frontal delta band and parietal theta band connectivity in MD children, future neurofeedback training or repetitive transcranial magnetic stimulation (rTMS) could attempt to modulate the activity in these frequency bands to improve their cognitive control, attention, and numerical processing abilities (Demirtas-Tatlidede et al., 2013; Enriquez-Geppert et al., 2017; Gruzelier, 2014; Zoefel et al., 2011). For the enhanced right temporal beta band connectivity in RD children, interventions might need to explore how to optimize this compensatory mechanism or strengthen left-hemisphere language processing functions through other means. Existing research has explored the application of neuro-modulation techniques in learning disorders; for example, neurofeedback training has been used to improve cognitive function in children with attention-deficit/hyperactivity disorder (ADHD), a condition often comorbid with learning disorders (Enriquez-Geppert et al., 2017). However, given the non-significant findings in this study regarding effective connectivity, these specific intervention targets require further validation in future research.

This study has several limitations. Firstly, the relatively small sample size limits the generalizability of the results and the statistical power, particularly in the effective connectivity analysis where no significant differences were found. Although this study controlled for IQ, attention, gender, and test scores as covariates in the statistical analysis, these general cognitive abilities often exhibit deficits in children with learning disorders (Moll et al., 2016; Peng and Fuchs, 2016), and their potential impact on brain network connectivity warrants deeper exploration. Future research should recruit larger samples and conduct a priori power analyses to ensure sufficient statistical power. It could also consider more complex statistical models to further decouple the effects specific to the disorder from those of general cognitive deficits.

Secondly, the RD and MD groups performed different tasks in this study, which complicates the direct comparison of learning disability-specific neural mechanisms. Different tasks activate distinct cognitive processes and neural networks; thus, observed connectivity pattern differences might reflect both task effects and disorder-specific effects. Future research should employ more rigorous experimental designs, such as having both groups of children complete both reading and mathematical tasks, or using resting-state EEG data for inter-group comparisons, to better separate task effects from disorder-specific effects.

Thirdly, while EEG possesses excellent temporal resolution, its spatial localization capability is limited, making it challenging to precisely identify the functional contributions of specific brain regions (e.g., specific sub-regions of the angular gyrus or intraparietal sulcus). For instance, the finding of “right temporal beta band synchrony” in this study still faces limitations in precise anatomical localization due to EEG's spatial resolution. Future research could consider integrating multimodal neuroimaging techniques, such as combining EEG with functional near-infrared spectroscopy (fNIRS) or fMRI, to compensate for EEG's limitations in spatial resolution and achieve spatiotemporally fused brain network analysis (Sun et al., 2013). Furthermore, the cross-sectional design of this study limits the verification of dynamic changes in neural plasticity. The study neither investigated the reorganization of connectivity patterns after training interventions nor established a dose-response relationship between neural changes and behavioral improvements (Keller and Just, 2016). Future research should adopt longitudinal designs to track brain network changes in children during development and evaluate the impact of interventions on the reorganization of these connectivity patterns, thereby establishing causal chains between neural changes and behavioral improvements (Keller and Just, 2016).

## 5 Conclusions

This study utilized task-related electroencephalography (EEG) to compare brain network functional and effective connectivity patterns across different frequency bands in children with Reading Difficulties (RD) during reading tasks and Math Difficulties (MD) during mathematical tasks. Our results indicate that, in terms of functional connectivity, the RD group exhibited significantly higher synchronization in the right temporal lobe's beta band than the MD group, while the MD group showed significantly greater connectivity in the frontal lobe's delta band and the parietal lobe's theta band. These findings suggest that RD children may engage in compensatory right-hemisphere involvement during reading, whereas MD children might face specific cognitive control and resource allocation challenges in frontal and parietal regions during mathematical tasks. However, no statistically significant differences were found between the two groups in effective connectivity. These findings provide preliminary evidence for understanding the neurophysiological basis of RD and MD and emphasize the critical role of task design and sample size in brain network research. Future studies should further explore the neural mechanisms of learning disabilities with larger sample sizes, more refined task designs, and multimodal imaging integration to lay a solid foundation for developing more targeted neuro-modulation intervention strategies.

## Data Availability

The original contributions presented in the study are included in the article/supplementary material, further inquiries can be directed to the corresponding author.
